# High LDL cholesterol and statin use were independently associated with lower eight-year mortality in a cohort free from terminal illness, cardiovascular disease, and diabetes at baseline

**DOI:** 10.3109/02813432.2014.869410

**Published:** 2014-03

**Authors:** Hans Thulesius

**Affiliations:** MD, GP, Associate Professor, Department of Clinical Sciences Malmö, Family Medicine Lund University, Kronoberg County Research Council Växjö,Sweden. E-mail: hansthulesius@gmail.com

## Cholesterol contrarians

It is well recognized that high cholesterol shows a strong correlation with cardiovascular disease (CVD) [1,2] and plays a major role in CVD risk-factor assessments [3]. Also, cholesterol-lowering drugs are increasingly recommended for primary prevention of CVD [4]. This knowledge is well established even if contrarian views exist suggesting bias in publication and reporting, and alternative mechanisms of action for statins [5–7]. In line with these contrasting explanations Bathum et al. showed reverse correlations between lipoprotein levels and mortality in a Danish observational study [8].

## A million person-years

The researchers compared data from several population-based registries linked with the unique Civil Personal Registration number for every Danish resident [8]. Between 1999 and 2008, the authors studied data from a cohort of almost 120 000 Danish people aged > 50 years without statin use, terminal illness, diabetes, or CVD at baseline with a median follow up of 8.5 years for age groups 50–70 years and 7.3 years for ages > 70 years. This produces data of almost one million person-years.

Hence, attempts at compensating for confounding factors were made by excluding people who had been prescribed statins the year before baseline, died within one year after baseline measurements, and those given a diagnosis of diabetes or CVD at any time before and until 14 days after baseline. Moreover, the results were presented by age, educational level, and gender-specific strata to compensate for these residual confounders.

## High cholesterol linked with lower mortality

Unpredictably, high LDL cholesterol (LDL-C > 3 mmol/L) was associated with reduced all-cause mortality with hazard-ratios ranging from 0.44 to 0.71 depending on age and gender. The association was strongest for men in the age group 50–60 years, showing a 56% lower risk of dying for men with LDL-C > 3 mmol/L than for those with LDL-C < 3 mmol/L. High levels of both total cholesterol (> 5 mmol/L) and HDL cholesterol (> 1 mmol/L) were associated with lower mortality, but hazard- ratios were not as low as for LDL-C levels.

## Statins linked with lower mortality

The cohort subgroup of 32% who had been prescribed statins on any occasion after baseline had lower all-cause mortality than the 68% without statin prescriptions, showing hazard-ratios of 0.28–0.69. Yet, this mortality reduction showed no correlation with baseline cholesterol levels and the interaction between high total cholesterol and statin use years was adjusted for in the analyses.

## Contradictory findings explained

The findings that both high LDL-C levels and the use of statins that reduce LDL-C were associated with low mortality are contradictory. So, what is the possible explanation for these unexpected discoveries? The authors first suggest that individuals with low cholesterol had severe diseases that lower cholesterol indicating a higher mortality in the group with low cholesterol. This could be the case even if the studied group did not include people who were terminally ill since people who died in the year following the baseline measurement were excluded from analysis. Thus, people with low cholesterol at baseline could have had more diseases connected with both low cholesterol and later higher mortality.

Second, there are other studies showing correlations between high total cholesterol and low mortality, especially in older age groups > 75 years [9]. In a Norwegian study, a U-shaped risk curve for total cholesterol suggested high mortality for both low and high levels of total cholesterol [10]. Another answer to the odd association could be that the people in the statin cohort adapted to a higher degree to a healthier lifestyle apart from taking statins – a change that would eventually reduce mortality.

## Cholesterol hypothesis questioned

In summary, Bathum et al. have undertaken an interesting observational study of lipoprotein levels, statin use, and mortality that adds to the growing number of publications questioning the mainstream cholesterol hypothesis of a clear correlation between high lipid levels and high mortality. At the same time, this study also provides data suggesting positive effects of statins on all-cause mortality. These positive effects of statins on survival were seen in all age groups and irrespective of baseline cholesterol levels.
